# Severe oxidative stress in sickle cell disease patients with uncomplicated *Plasmodium falciparum* malaria in Kampala, Uganda

**DOI:** 10.1186/s12879-019-4221-y

**Published:** 2019-07-09

**Authors:** Saad Mahjub Atiku, Nabukeera Louise, Dennis M. Kasozi

**Affiliations:** Department of Biochemistry and Sports Science, School of Biosciences, College of Natural Sciences, Makerere University Kampala, P.O. BOX 7062 Kampala, Uganda

**Keywords:** Sickle cell disease, Malaria, Biomarkers of oxidative stress, Malondialdehyde, Catalase, Uganda

## Abstract

**Background:**

Oxidative stress plays a vital role in the pathogenesis of both Sickle Cell Disease (SCD) and *Plasmodium falciparum* malaria. However, there are limited studies on the effect of *P. falciparum* malaria infection on oxidative stress in SCD patients.

**Methods:**

A cross-sectional study was undertaken to compare levels of biomarkers of oxidative stress in isolates from SCD patients with uncomplicated *P.falciparum* malaria. The biomarkers namely: malondialdehyde (MDA), reduced glutathione (GSH), catalase (CAT) and glutathione peroxidase (GPx) were determined in plasma samples from SCD malaria positive, malaria positive, SCD malaria negative and healthy control participants. The genetic diversity of *P.falciparum* was determined by nested polymerase chain reaction of merozoite surface protein-2 (MSP-2) gene.

**Results:**

Out of 207 participants, 54 (26%) were SCD malaria positive, 51 (24%) malaria positive, 51 (24%) SCD controls and 51 (24%) healthy control individuals. The mean concentration of MDA was significantly higher in SCD malaria positive than SCD controls (*P* < 0.0001). In contrast, the mean concentration of GSH (*P* < 0.0001) and GPx (*P* < 0.0001) were significantly lower in SCD malaria than SCD controls. Although not significantly different, the mean concentration of MDA was higher (*P* = 0.0478), but the geometric mean parasite density (*P* = 0.2430) and multiplicity of infection (*P* = 0.3478) were lower in SCD malaria samples than in malaria samples. The most prevalent MSP2 allelic family was IC3D7 in SCD malaria (72%) and Malaria (76%) samples. The biomarkers of oxidative stress were not significantly different between IC3D7 and FC27 allelic families of MSP2.

**Conclusion:**

We identified severe oxidative stress in Sickle cell disease patients with uncomplicated *P.falciparum* malaria.

## Background

Sickle cell disease (SCD) and Malaria are co-endemic in Africa. SCD is a hemoglobinopathy caused by a single mutation in the β-globin chain resulting in the substitution of valine for glutamic acid at the sixth amino acid position [1]. Worldwide, an estimated 312,000 children are born with SCD annually 75% of these in Sub Saharan Africa [2]. In Uganda it is estimated that 15,000 babies are born with SCD and of these 50–80% die annually before 5 years [3]. With Uganda having the third highest number of *Plasmodium falciparum* infections in sub-Saharan Africa [4], malaria is considered a significant cause of this SCD mortality [5].

Oxidative stress is plays a vital role in the pathophysiology of both SCD [6] and malaria [7]. In SCD, oxidative stress results from increased intravascular hemolysis, ischemia-reperfusion injury, and chronic inflammation [6]. In response to *P.falciparum* infection, phagocytes generate large amounts of reactive oxygen species (ROS) and reactive nitrogen species (RNS) causing an imbalance between oxidizing species and antioxidants [7]. This imbalance triggers oxidative stress, which is an important mechanism of human hosts in response to infection and can lead to the death of the parasites [8] although it may also lead to cell and tissue damage [9]. The host enzymatic and nonenzymatic antioxidant cellular defense system plays a key role in protecting biological systems from ROS by regulating the production of free radicals and their metabolites [10]. The primary antioxidant enzymes against superoxide radicals include superoxide dismutase (SOD), catalase (CAT), and glutathione peroxidase (GPx) [10]. The major non-enzymatic antioxidant cellular defense systems comprise glutathione, vitamin C and Vitamin E [10]. Limited studies have been undertaken on oxidative stress in SCD patients in Sub-Saharan Africa. Malondialdehyde (MDA) which is a product of lipid peroxidation is an important marker in the evaluation of oxidative stress [9, 10]. Recently, a study in Cameroon showed increased MDA in SCD patients compared to healthy individuals [11]. However, no study has evaluated oxidative stress in SCD patients with *P.falciparum* malaria in Uganda.

In addition, to date there is very limited information on the impact of oxidative stress on the genetic diversity of *P.falciparum*. Strains of *P.falciparum* are distinguished by highly polymorphic markers, in particular the merozoite surface protein 2 (MSP2) [12]. Recently, significant disparity in allele frequencies of K1, MAD20, RO33 of MSP 1 were found in SCD patients with malaria in Nigeria [13]. Yet, by far MSP2 is more polymorphic and has the higher heterozygosity values than MSP1 [12]. However, no studies have been undertaken on the distribution of IC3D7 and FC27 the allelic families of MSP2 in SCD patients in Sub Sahara Africa.

It is important to evaluate the oxidative status of SCD patients with *P.falciparum* malaria. This evidence would be relevant in the improved management of malaria in SCD. The aim of this study was to characterize oxidative stress in SCD patients with *P.falciparum* malaria.

## Methods

### Study design

This was a cross-sectional study done between November 2015 and April 2016. Ethical approval was obtained from Mulago National Referral Hospital Ethics Committee. The inclusion criteria were: blood samples of SCD patients confirmed positive for *P. falciparum*, SCD patients who were malaria negative, malaria positive and healthy controls. SCD was confirmed by Hb electrophoresis on cellulose acetate membrane. The **exclusion criteria** were: Samples from patients with chronic and complicated conditions including severe or complicated malaria, severe anaemia (Hb < 7 g/dl) and other infections like HIV and on any treatment were excluded. Samples were obtained from participants after informed consent from their parents or guardians. All patients were treated as per the national antimalarial guidelines. To diagnose *P.falciparum* by microscopy, thick blood films of every participant were made and stained using 10% Giesma stain. The parasite density was expressed as number of parasites per microlitre (μL) of blood, assuming a WBC count of 8000 leucocytes per μL of blood.

### Blood sample collection

Venous blood (3 mL) was collected from each participant into a purple top tube containing Ethylene Diamine Tetra Acetate (EDTA). All the blood samples were centrifuged at X3000rpm for 10 min. The plasma if not analyzed immediately was stored for not longer than 48 h in a freezer (− 20 °C) for the MDA and GSH assays. A total of 207 samples were collected from Mulago Hospital, Kampala Uganda. Out of these 105 samples were from Sickle Cell Clinic of Mulago hospital, 54 were *P.falciparum* malaria positive and 51 negative (sickle cell control). Fifty one *P.falciparum* malaria positive were obtained from Malaria Lab at Assessment Centre of Mulago Hospital. Fifty one samples for Healthy control group were obtained from the Nakasero Blood Bank and tested within 48 h after collection. In addition, RBCs were lysed with ice cold water and centrifuged at 5000 rpm for 10 min at 4 °C to obtain the clear lysate that was used for the CAT and GPx assays. The analysis for the biomarkers of oxidative stress was done at the Biochemistry Department, Makerere University.

### Preparation of dry blood spots (DBS)

*P. falciparum* positive Blood samples (50 μL) were placed on 903 filter papers (Fitzco, Minneapolis, MN) labeled, air dried and then placed in plastic bags. The DBS were stored at ambient temperature for MSP 2 genotyping.

### Malondialdehyde (MDA)

MDA was determined using the Thiobarbituric acid reactive substances (TBARS) assay which is based on the reaction between MDA and 2-thiobarbituric acid, a chromogenic reagent to form a chromophore with absorbance maximum at 532 nm [14]. In the assay, 300 μl of the sample plasma was mixed with 200 μL of Trichloro acetic acid (TCA)/ Thiobarbituric acid (TBA) solution containing 15 g of TCA and 0.67 g TBA in 50 ml of 0.25 M HCl. Next 300 μl of 1 M HCl was added to this mixture and then heated at 100 °C for 60 min. The mixture was cooled on ice for 20 min and centrifuged at 3000 rpm for 2 min. To 350 μl of the supernatant was then added 300 μl of 3 M NaOH and vortexed. The mixture was spinned again at 1300 rpm for 1 min before reading the absorbance at 540 nm within 10 min.

### Level of the reduced glutathione (GSH)

The determination of Glutathione levels in the plasma was performed as described [15]. The assay is based on the oxidation of GSH by 2-nitrobenzoic acid (DTNB) to form the yellow chromophore 5′-thio-2-nitrobenzoic acid (TNB), with absorbance at 412 nm. Briefly, 200 μL of 10% solution of TCA was added into 1.5 ml Eppendorf tube followed by 100 μL of freshly thawed plasma. The mixture was then vortexed, centrifuged at 4000 x g for 10 min and the supernatant separated. To 200 μL of the supernatant 700 μL of 400 mM Tris-HCl buffer, pH 8.9 was added followed by 100 μL of 2.5 mM DTNB dissolved in 40 mM Tris-HCl buffer, pH 8.9 and the mixture was then incubated at room temperature for 10 min. The optical densities (O.Ds) of the samples/standards and the blank (made of DTNB instead of sample/standard) at 412 nm were measured. The O.Ds were then used to determine the concentration of GSH using the calibration graph of the standards.

### Activity of glutathione peroxidase (GPx)

This was measured indirectly using a modification of Ursini et al., [16] where 100 μl of 2 mM GSH solution was added to 150 μl of 0.1 M phosphate buffer pH 7.4. Then 50 μl of 10 mM sodium azide and 1 mM H_2_O_2_ was added to this mixture followed by 150 μl of the sample lysate and the mixture was then vortexed. A blank made of 400 μl, 50 μl and 50 μl of 0.1 M phosphate buffer pH 7.4, 10 mM sodium azide and 1 mM H_2_O_2_ respectively while the reference standard consist of 300 μl, 100 μl, 50 μl and 50 μl of 0.1 M phosphate buffer pH 7.4, 2 mM GSH solution, 10 mM sodium azide and 1 mM H_2_O_2_ respectively. The mixtures were then incubated at 37 °C for 15 min in a water bath, 250 μl of 5%TCA was added and the mixture spinned at X2000rpm for 5 min. Next 25 μl of the supernatant was then transferred into a 96 well plate containing 50 μl of the buffer and 175 μl of DTNB. The plate was shaken for 1 min and absorbance read at 405 nm using an ELISA plate reader.

### Activity of catalase

The activity of catalase was determined using a modification of a colorimetric assay as described [17] which determines the H_2_O_2_ content of erythrocyte lysates. Briefly, 200 μl of 0.01 M phosphate buffer was added to 300 μl of 0.2 M hydrogen peroxide and to this mixture 20 μl of diluted sample lysate (1:10 with 0.01 M phosphate buffer pH 7.0) was added. This mixture was then swirled, incubated at room temperature for 1 min and 500 μl of 5% solution of K_2_Cr_2_O_7_ with glacial acetic acid (1,3 by volume) added. The mixture was vortexed and incubated at 100 °C for 10 min before reading absorbance at 580 nm.

### Extraction of DNA

Total genomic DNA was extracted from dried blood spots (DBS) on paper cards (Fitzco, Minneapolis, MN) by punching 3 dB into a 1.5 ml tube. To this 300 μL of methanol was added to completely immerse the DBS and the content to be incubated at room temperature for 15 min. The methanol was then discarded carefully and the retained DBS dried in the tubes for 15 min.100 μL of distilled water was added to the DBS and the content heated at temperatures of between 95 and 100 °C for 15 min while occasionally vortexing to increase yield. The extracted DNA was then transferred into a 25 μL PCR reaction tube or frozen for later use in analysis of the MSP2 gene.

### Genotyping MSP2 of *P.falciparum* isolates

PCR was a two-step reaction involving a primary and nested PCR reaction in which amplification of the entire *msp2* gene was followed by targeting the allelic families of IC/3D7 and FC27 [12]. The sequence of the oligonucleotide primers and genomic DNA from cloned laboratory strains which were used as positive controls are shown in Table 1. Both primary and nested PCR was used to amplify the polymorphic repetitive region (block 3) of *msp2* where in the primary reaction, primer pairs corresponding to conserved sequences spanning the polymorphic region of the *msp2* genes was used and in the second nested reactions, specific primer pair was then used to determine the presence of allelic variants from the FC27 and the IC/3D7 families of the *msp2* repeats.

**Table 1:** Primers sequences and annealing temperature for nested PCR of MSP2

PCR round	Primer name.	Primer Sequence.(5′ → 3′)	Annealing temp (°C)	Positive Control	Allelic Family
Primary	1	ATGAAGGTAATTAAAACATTGTCTATTATA	55		
	4	ATATGGCAAAAGATAAAACAAGTGTTGCTG	55		
Secondary	A1	GCAGAAAGTAAGCCTTCTACTGGTGCT	55	3D7	IC3D7
	A2	GATTTGTTTCGGCATTATTATGA	55		
	B1	GCAAATGAAGGTTCTAATACTAATAG	55	HB3	FC27
	B1	GCTTTGGGTCCTTCTTCAGTTGATTC	55		

*PCR* Polymerase chain reaction, *MSP2* Merozoite surface protein 2

Amplifications was performed in Thermocycler (Biometra, T3000) with a total volume of the reaction to be 25 μL which was composed of 23 μL of a master mix (160.5 μL of distilled H_2_O, 5 μL each of primer 1 and primer 2, 25 μL of buffer, 25 μL of dNTPs, 7.5 μL of MgCl_2_ and 2 μL of Taq Polymerase) and 2 μL of the samples and the controls. Thus 23 μL of the master mix was added to each well of a standard PCR tube corresponding to a sample or a control followed by 2 μL of the sample or control and pressure applied for tight sealing. The thermocycler which was programmed to perform the primary PCR amplification of *msp2* genes as follows; an initial step of 95 °C for 5 min followed by 30 cycles of 95 °C for 1 min, 55 °C for 2 min, 72 °C for 2 min, and a final extension of 72 °C for 5 min..

To identify the positive samples, the nested *msp2* amplification product was separated in a 2% ethidium bromide-stained agarose gel. Briefly 3 μL of loading dye was mixed with 15 μL of the PCR product and carefully loaded into an appropriate well on a gel. The first and last wells were used for the DNA size standards (50 bp). PCR products were loaded between the first and last wells. Agarose gel electrophoresis was carried out for 30 min at 85–90 V. Once the gel had run sufficiently, it was photographed digitally using UV light and saved in a format compatible with GelCompar II, a gel processing software for band sizing and molecular weight calculations. The ratio of the number of total fragments to the number of positive samples was taken as the MOI [18, 19].

### Data analysis

The results were analyzed using GraphPad Prism 5.03 software. One way analysis of variance (ANOVA) was used to assess significant differences between the groups. The linear association between parasite density and biomarkers of oxidative stress were analyzed by Pearson correlation coefficient. Statistical significance was defined as *P* < 0.05.

## Results

### Baseline characteristics

Out of 207 participants, 54 (26%) were SCD malaria positive, 51 (24%) malaria positive, 51 (24%) SCD malaria negative and 51 (24%) healthy control individuals. The mean age (years) of SCD malaria positive, malaria positive, SCD malaria negative and healthy control individuals were 14.1 (SD 10.2), 12.6 (SD 12.2), 14.8 (SD13.6) and 22 (SD 7.2) respectively. The percentage of females in SCD malaria positive was 44.4% (24/54), malaria positive 47.1% (24/51), SCD malaria negative 45.1% (23/51) and healthy control individuals 54.9% (28/51). The mean hemoglobin concentration (g/dL) of SCD malaria positive, malaria positive, SCD controls and healthy control individuals were 8.4 (SD 1.9), 12.9 (SD 1.6), 9.2 (SD 1.5) and 14.2 (SD 1.4) respectively. The geometric mean parasite density (GMPD) was lower in SCD malaria (16,800, 95%CI 12,131–23,266 parasites/μL) than in the malaria samples (19,306, 95%CI 16,706–22,310 parasites/μL) but the difference was not significant (Unpaired t test, *P* = 0.2430).

### Markers of oxidative stress in sickle cell malaria patients and control groups

**Fig. 1:**
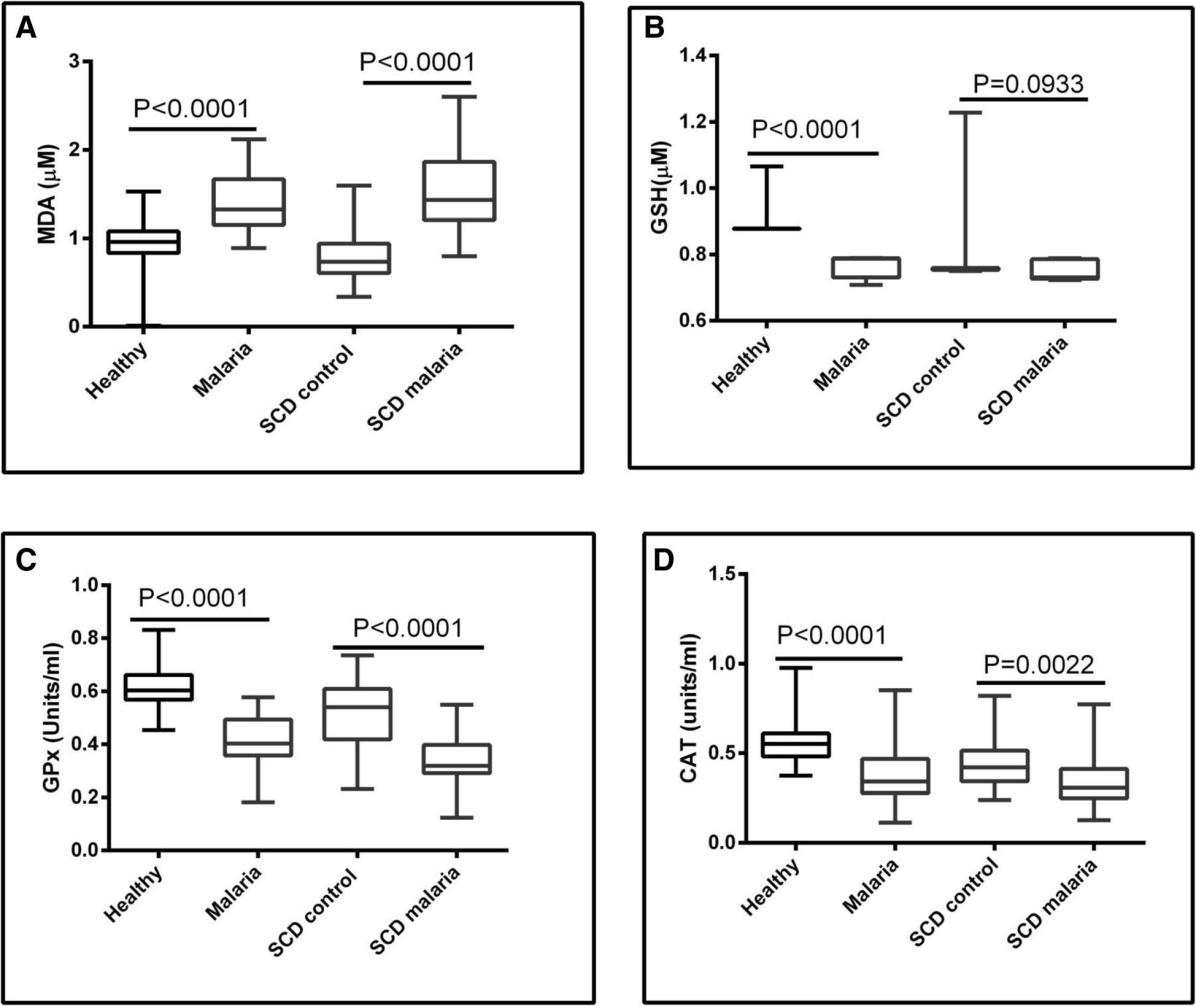
Variation in Biomarkers of Oxidative stress. The concentration of MDA (**a**) increased but the concentration of GSH (**b**) and activity GPx (**c**) as well as that of CAT (**d**) decreased between healthy control and malaria as well as between the SCD control and SCD malaria group. The mean MDA, GSH, GPx and CAT were significantly different (ANOVA, *P* < 0.001). MDA: Malondialdehyde; GSH: reduced glutathione; GPx: glutathione peroxidase; CAT: Catalase

The mean levels of markers of oxidative stress namely MDA, GSH, GPx and CAT are shown in Table 2. The variations in the levels of markers of oxidative stress are shown in Fig. 1. The mean concentration of MDA was significantly higher in isolates from SCD malaria positive compared to SCD control group (*P* < 0.0001). By contrast, mean levels of GPx (*P* < 0.0001) and CAT (*P* = 0.0022) were significantly higher in SCD control group than SCD malaria.

**Table 2:** Markers of oxidative stress

Group	MDA ± SD (μM)	GSH ± SD (mM)	GPx ± SD (Units/ml)	CAT± SD (Units)
SCD malaria	1.58 ± 0.456	0.726 ± 0.028	0.344 ± 0.0802	0.355 ± 0.156
Malaria	1.39 ± 0.325	0.767 ± 0.031	0.412 ± 0.0962	0.383 ± 0.164
SCD control	0.81 ± 0.300	0.766 ± 0.063	0.530 ± 0.0115	0.436 ± 0.129
Healthy	0.88 ± 0.277	0.929 ± 0.083	0.615 ± 0.0824	0.562 ± 0.108
*P-value* ^a^	0.0001**	0.0001**	0.0001**	0.0001**

*MDA* Malondialdehyde, *GS* Reduced Glutathione, *CAT* Catalase, *GPX* Glutathione peroxidase

**Statistically significant, ^a^ANOVA

Although the mean level of GSH was also higher in SCD control group than SCD malaria, the difference was not statistically significant (*P* = 0.0933). The mean concentration of MDA was significantly higher in isolates from malaria positive compared to healthy control group (Table 1, *P* < 0.0001). Conversely, mean levels of GSH (*P* < 0.0001), GPx (*P* < 0.0001) and CAT (P < 0.0001) were significantly higher in the healthy control group than in the malaria group.

There was no significant difference (*P* = 0.0905) in mean level of MDA between SCD control group and the healthy control group. On the contrary, mean levels of GSH (*P* < 0.0001), GPx (*P* < 0.0001) and CAT (*P* < 0.0001) were significantly higher in healthy control group than the SCD control group. Although not significantly different, the mean level of MDA was also higher in SCD malaria group than malaria group (*P* = 0.0478). In contrast, mean levels of GSH (*P* = 0.0052), and GPx (*P* = 0.0002) were significantly higher in malaria group than the SCD malaria group. The mean level of CAT was also higher in malaria group than SCD malaria group, but the difference was not statistically significant (*P* = 0.3085).

### Genetic diversity, allelic frequency and multiplicity of infection

**Fig. 2:**
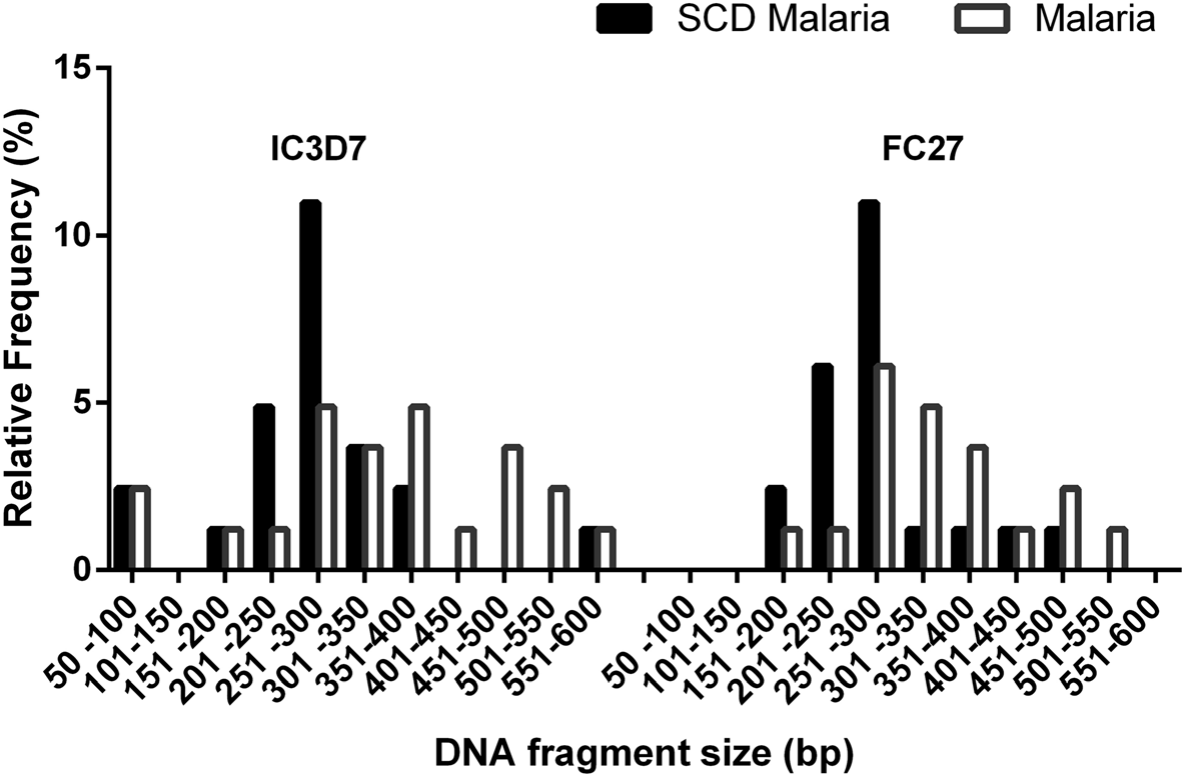
Allelic families of MSP2. Comparison of the distribution of the IC/3D7 and FC27 allelic families between isolates from malaria and SCD malaria patients

Out of 50 isolates analysed, 25 (50%) malaria positive and 25 (50%) SCD malaria positive isolates were successfully genotyped for the detection of MSP2 alleles. The fragement sizes for IC3D7 and FC27 in SCD malaria and malaria samples are shown in Fig. 2. The most prevalent MSP2 allelic family was IC3D7 in SCD malaria (72%) and Malaria (76%) samples. For the SCD malaria positive isolates, 24% were positive for IC3D7 allelic family, 20% for the FC27 allelic family, 48% for both allelic families. In the *P.falciparum* malaria positive isolates, 36% were positive for the 3D7 allelic family, 20% for the FC27 allelic family, 40% for both allelic families. In the SCD malaria positive isolates, 14 different alleles were identified with 7 alleles belonging to each of the allelic families of IC3D7 and FC27. In the malaria positive isolates, 18 alleles in total were identified with 10 for the 3D7/IC family (50-600 bp) and 8 for the FC27 family (150-550 bp) The Multiplicity of infection (MOI) was lower in the SCD malaria positive isolates (1.67) than in the malaria positive (1.75) isolates but the difference was not statistically different (*P* = 0.3478).

### Parasite density and biomakers of oxidative stress

**Fig. 3:**
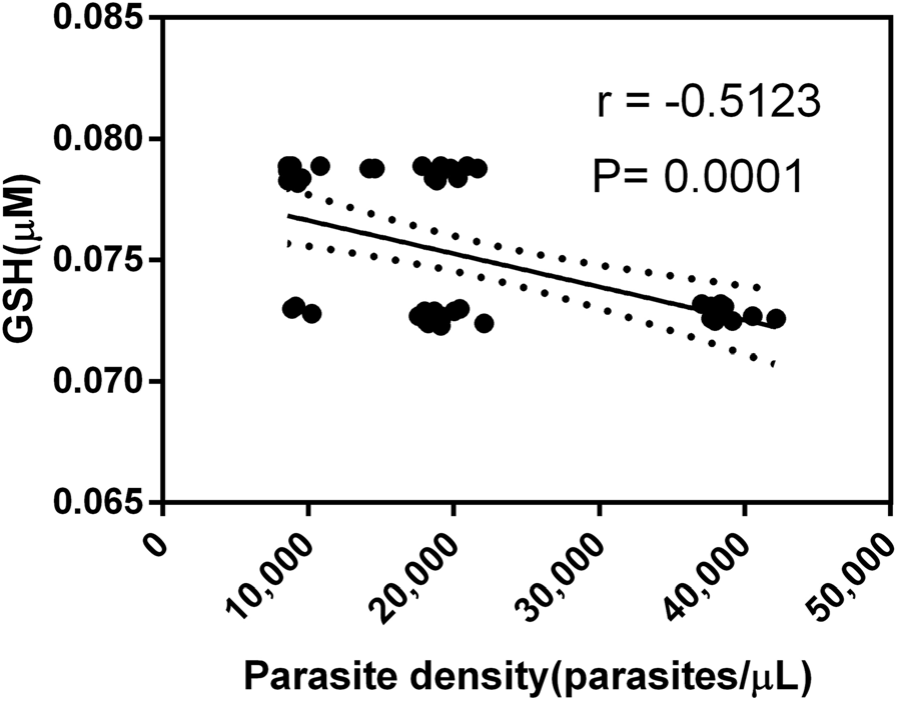
Correlation between Parasite density and Glutathione. In the SCD patients with malaria, the correlation between parasite density (parasites/μL) and glutathione (μM) was significant (Pearson’s r = − 0.5123, *P* = 0.0001)

Table 3 shows the Pearson correlation coefficents between parasite density and levels of biomarkers of oxidative stress. There were no significant correlations between parasite density and MDA, activity of GPx and CAT in SCD patients with malaria except for GSH with parasite density (Fig. 3). In the normal patients with *P. falciparum malaria*, parasite density with MDA or GPx had a positive correlation which was not statistically significant. However, GSH and CAT had negative correlation but was also not statistically significant.

**Table 3:** Correlation of Parasite Density with Biomarkers of oxidative stress

	MDA (μM)	GSH (μM)	GPx (Units/ml)	CAT (U/mg prot)
SCD malaria				
n	51	51	51	51
Pearson r	0.07110	− 0.5123	−0.02336	0.1780
*P* value	0.6237	0.0001	0.8721	0.2162
R^2^	0.005055	0.2625	0.0005455	0.03168
Malaria				
n	50	50	50	50
Pearson r	0.1206	−0.1142	0.1198	−0.01226
*P* value	0.4040	0.4295	0.4074	0.9327
R^2^	0.01455	0.01305	0.01435	0.0001503

Pearson’s **r** values from scatter plots. With *n* = 50, MDA is malondialdehyde; GSH is reduced glutathione; GPx is Glutathione peroxidase; CAT is catalase

### Malondialdehyde and other biomarkers of oxidative stress

Table 4 shows pearson correlation coefficents between MDA and other biomarkers of oxidative stress. In the SCD malaria group, malaria group and SCD control group, the correlations between MDA with GPx were positive but not statistically significant. In constrast, in the healthy control group there was no correlation between MDA with GPx. In all groups, there were no correlations between MDA with GSH and CAT.

**Table 4:** MDA and other Biomarkers of Oxidative stress

Biomarker	GSH	GPx	CAT
SCD Malaria n	51	51	51
Pearson r	−0.2500	0.2893	−0.1456
*P* value (two-tailed)	0.0769	0.0395	0.3081
R^2^	0.06249	0.08369	0.02119
Malaria			
Pearson r	0.01729	0.07464	0.1865
*P* value (two-tailed)	0.9013	0.5917	0.1769
R^2^	0.0002988	0.005571	0.03479
SCD control			
Pearson r	−0.05931	−0.3836	0.07885
*P* value (two-tailed)	0.6793	0.0055	0.5823
R^2^	0.003518	0.1472	0.006217
Healthy control			
Pearson r	0.01596	0.0278	−0.01898
*P* value (two-tailed)	0.9115	0.8464	0.8948
R^2^	0.0002547	0.0007729	0.0003601

*MDA* malondialdehyde, *GSH* reduced glutathione, *GPx* Glutathione peroxidase (a negative r value that is significant), *CAT* catalase

### Biomarkers of oxidative stress and MSP2 alleles

No significant differences were observed between MDA, GSH, GPx and CAT between IC3D7 and FC27 in SCD malaria and malaria samples. Table 5 shows the relationship between the oxidative stress biomarkers and MSP2 allele families.

**Table 5:** Oxidative stress Biomarkers with MSP2 alleles

	MDA (μM)	GSH (μM)	GPx (Units/ml)	CAT (U/mg prot)
SCD Malaria (n)	25	25	25	25
IC3D7	1.57 ± 0.09	0.74 ± 0.01	0.38 ± 0.01	3.67 ± 0.29
FC27	1.56 ± 0.08	0.75 ± 0.01	0.34 ± 0.02	3.31 ± 0.21
*P* value ^a^	0.9420	0.3705	0.0959	0.3298
Malaria				
IC3D7	1.43 ± 0.06	0.75 ± 0.01	0.43 ± 0.02	2.87 ± 0.19
FC27	1.46 ± 0.08	0.74 ± 0.01	0.41 ± 0.02	3.25 ± 0.31
*P* value ^a^	0.7571	0.6099	0.3689	0.3041
*P* value ^b^	0.2382	0.3859	0.0400**	0.0282**
*P* value ^c^	0.3813	0.6112	0.0267**	0.8896

MDA is malondialdehyde; GSH is reduced glutathione; GPx is Glutathione peroxidase; CAT is catalase. IC3D7 and FC27 are allele families of MSP2, *** Statistically significant. ^a^Between IC3D7 and FC27 in each group; ^b^ Between IC3D7 in SCD malaria and malaria groups; ^C^ Between FC27 in SCD malaria and malaria groups

## Discussion

Our study for the first time, characterized significantly elevated oxidative stress in patients having the SCD with uncomplicated *P.falciparum* malaria. This work assessed biomarkers of oxidative stress including malondialdehyde (MDA), reduced glutathione (GSH), glutathione peroxidase (GPx) and catalase (CAT) in isolates from SCD patients with *P.falciparum* malaria.

In SCD, the rate of polymerization of sickled hemoglobin (HbS) increases with increase in its concentration. This increase in polymerization results into elevated hemolysis and hence the release of cell-free heme released after autoxidation [19]. During the reperfusion period, heme auto-oxidizes in the presence of oxygen into methemoglobin and superoxide. Whereas the normal hemoglobin (HbA) can convert them to harmless byproducts, the HbS produces H_2_O_2_ which when exposed to methemoglobin, decomposes hemoglobin to form ferrous (Fe^2+^) [20]. Hydrogen peroxide then reacts with the ferrous (Fe^2+^) formed to liberate the more reactive and harmful radical of hydroxyl (**•**OH) [20]. This implies that HbS liberates more concentrations of superoxide, H_2_O_2_, and **•**OH than HbA and hence increased oxidative stress.

The current study showed higher concentration of MDA in the malaria positive samples than the negative samples indicating increased oxidative stress. These findings are in agreement with previous results [21, 22]. These results may be explained by the fact that the malaria parasite is capable of generating reactive oxygen species within the erythrocytes [6, 7]. Furthermore, increased oxidative stress may be explained as arising from the ROS produced from immune activation against the malarial parasite an antimicrobial action [23].

The difference in mean level of MDA between SCD control group and healthy control group, was not statistically significant (*P* = 0.0905). A previous study in Cameroon showed that MDA in SCD patients was significantly higher than in healthy individuals [11]. This discrepancy in results may be due hydroxyurea medication used by the sickle cell disease patients. Hydroxyurea has been shown have antioxidant property of decreasing the levels of lipid peroxidation [24].

GSH is a vital antioxidant in redox homeostasis. The increased oxidative stress in both the Sickle cell disease [25] and malaria [7] elevates the demand for GSH. Several authors have reported an irreversible loss of GSH especially when it acts as a de-toxicant [26, 27]. The results obtained in this study agree with the above previous work with reduction in GSH as oxidative stress increases.

GPx is an important enzymatic antioxidant found in the cytosolic and mitochondrial as well as in the peroxisomal compartments in some cells [28]. GPx plays a vital role in the elimination of both hydrogen peroxide and lipid type of peroxides. Several authors attribute the total elimination of hydrogen peroxide to GPx [28, 29]. Several other authors also report reduced GPx activity in erythrocytes under oxidative stress which is in agreement with the findings in this work where activity of GPx in malaria positive isolates is lower than the controls with the activity.

Catalase plays a vital role in the decomposition of hydrogen peroxide to water and oxygen [30]. In this study there is a significant decrease in catalase activity in the malaria positive isolates compared to controls which is in total agreement with them. Furthermore, this study also reports a further decrease activity of catalase in isolates with SCD disease than the healthy normal controls. Similar to previous studies, several authors have reported a decrease in the activity of erythrocyte catalase under oxidative stress [30, 31].

In the current study, there was a non-significant trend of a lower parasitemia in SCD malaria compared to malaria patients without SCD. This may be attributed to the enhanced phagocytosis of ring-parasitized SCD RBC compared to normal RBCs [32]. This is consistent with previous study in Dar-es-Salaam, Tanzania [33]. This finding may be interpreted by the fact that greater oxidative stress (higher MDA levels) damages erythrocytes and may also directly or indirectly damage the parasites resulting in lower parasitemia. Another explanation may be that MDA leads to ineffective erythropoiesis. Furthermore, parasite density had positive correlation with MDA but this was not statistically significant. This finding may be explained by the fact that MDA is not the only end product of the destructive effects of ROS but also 4-hydroxynonenal [34]. In addition, no significant correlations were found between parasite density and MDA with other biomarkers of oxidative stress. Several components of the host enzymatic and nonenzymatic antioxidant cellular defense system are synergistically involved in protecting biological systems from ROS [7].

In this study, the prevalence of the IC3D7 was higher than the FC27 allelic family as prevoiusly reported in malaria patients without SCD [35, 36]. However, findings in this study indicate a lower MOI than previously shown in Arua, Uganda [37] and Gezira state Sudan [38] though in consistent with that in Malaysia [39]. In Uganda, several genotypic studies of *P. falciparum* have been done in malaria patients with normal hemoglobin and sickle cell trait. A previous study in Western Uganda found no significant influence of sickle cell trait on the distribution of FC27 and IC3D7 alleic famillies [40].

Of note, no studies have investigated genetic diversity of *P.falciparum* strains in SCD patients with malaria in Uganda. Using a far more polymophic marker MSP2, this study showed predominance of IC3D7 family but lower MOI and no association with biomarkers of oxidative stress. Recently, a significant disparity in MSP1 genotypes and allele frequencies (K1, 91.8%, MAD20, 32.4%; RO33, 18.9%) were found in SCD patients with malaria in Nigeria [13]. The lower MOI may be due to severe oxidative stress in SCD malaria. In constrast, a much lower overall MOI of 1.43 was found SCD patients with malaria in Nigeria [13]. These findings could also explained that oxidatives stress damages not only erythrocytes but also malaria parasites. Furthermore, the study found no significant differences were observed between MDA, GSH, GPx and CAT between IC3D7 and FC27 in SCD malaria and malaria samples. It can be concluded that infection by *P.falciparum* causes oxidative stress regardless of parasite strain.

This study had several limitations. First the study had a small sample size for each of the four groups (~ 50 each) which may have limted our power to identify certain effects. Secondly, TBARS method assay is known to have limitations in that it measures many different aldehydes and decomposition products from H2O2 which may translate into problems with specificity and variability of data. Thirdly, reticulocyte counts were not determined which could have provided useful information for preliminary assessment of hemolytic or dyserythropoietic anemia. More studies are needed to investigate these aspects. Nevertheless, our study provides novel insights into the effects of malaria on oxidative stress in SCD patients (HbSS).

## Conclusion

This study showed increased lipid peroxidation and reduced antioxidant levels in the SCD+/ malaria isolates than the SCD controls. Although not significantly different, the mean GMPD was lower in SCD+/ malaria patients than SCD−/malaria patients. Thus, erythrocytes from SCD patients are subjected to a double oxidative stress, the first stress due to the mutation and secondly stress induced by the developing *P.falciparum* parasites [32]. These results confirm severe oxidative stress in SCD patients with uncomplicated *P.falciparum* malaria.

## Data Availability

The datasets used and/or analyzed during the current study are available from the corresponding author on reasonable request.

## Abbreviations

CAT: Catalase
DTNB: 2-nitrobenzoic acid
GMPD: Geometric mean parasite density
GPX: Glutathione peroxidase
GSH: Reduced glutathione
HbAA: Normal hemoglobin
HbAS: Sickle cell trait
HbSS: Sickle cell patients
MDA: Malondialdehyde
MSP2: merozoite surface protein 2
RNS: Reactive nitrogen species
ROS: Reactive oxygen species
SCD: Sickle cell disease
SD: Standard deviation
TBA: Thiobarbituric acid
TCA: Trichloroacetic acetic acid
TNB: 5′-thio-2-nitrobenzoic acid
